# Supplementary material for: Aqueous proton-selective conduction across two-dimensional graphyne
Source: Nat Commun. 2019 Mar 11;10:1165. doi: 10.1038/s41467-019-09151-8 (PMC6412031; doi:10.1038/s41467-019-09151-8)
Supplement: Supplementary file 1 — Supplementary Information [file 41467_2019_9151_MOESM1_ESM.pdf]

Aqueous proton-selective conduction across two-dimensional graphyne

Supplementary Information

Le Shi[1], Ao Xu[1], Ding Pan[2,3], Tianshou Zhao[*,1]

1. HKUST Energy Institute, Department of Mechanical and Aerospace Engineering, The Hong Kong University of Science and Technology, Hong Kong, China

2. Department of Physics and Department of Chemistry, The Hong Kong University of Science and Technology, Hong Kong, China

3. HKUST Fok Ying Tung Research Center, Guangzhou, China

*Corresponding author. Tel.: (852) 2358 8647 E-mail: metzhao@ust.hk (T.S. Zhao)

Supplementary Note 1

For proton penetration across graphyne (*n*=1 and *n*=2), spring force with force constant of 15000 KJ (mol·Å)$^{-1}$ was applied once the O-H distance in a predefined water molecule exceeds 1.4 Å or the predefined proton moves more than 4 Å away from graphyne. As the index of excess proton is specified and won't change due to the constraints applied, the collective variable (CV) was defined as the distance *L* between the position of the specified proton $\mathbf{r}_p(t)$ and graphyne. It reads:

$$L = r_p(z) - g(z) \tag{S1}$$

where $r_p(z)$ and $g(z)$ means the position of proton and graphyne in *z* direction. For proton penetration across graphyne (*n*=2, *n*=3 and *n*=4) without constraints, proton hops between water molecules through Grotthuss mechanism. The CV is defined as the distance *L* between the oxygen atom in the hydronium ion $\mathbf{r}_O(t)$ and graphyne,

$$L = r_O(z) - g(z) \tag{S2}$$

where $r_O(z)$ can be calculated using [1, 2]:

$$r_O(z) = \frac{\sum_{i\in\{O_w\}} z_i e^{\lambda n_i}}{\sum_{i\in\{O_w\}} e^{\lambda n_i}} \tag{S3}$$

where z_i is the *z* position of waters' or hydronium's oxygen *i*, λ is a large number, and $\{O_w\}$ refers to all oxygen atoms in the simulation system. The variable n_i is the hydrogen coordination number:

$$n_i = \sum_{j\in\{H_w\}} n(r_{ij}) \tag{S4}$$

where $\mathrm{n}(r_{ij}) = \frac{1-(\frac{r_{ij}}{r_0})^6}{1-(\frac{r_{ij}}{r_0})^{12}}$, r_{ij} is the distance between oxygen atom *i* and hydrogen atom *j*, and $\{H_w\}$ refers to all hydrogen atoms in the simulation system. The variable r_0 is set to be 1.25 Å. The variable n_i is very close to 3 when *i* is the oxygen atom of the hydronium ion and 2 in the case of a water molecule.

For methanol penetration across graphyne (*n*=3 and *n*=4), the CV was chosen as the distance *L* between the carbon atom in the methanol molecule $\mathbf{r}_C(t)$ and graphyne:

$$L = r_C(z) - g(z) \tag{S5}$$

where $r_C(z)$ and $g(z)$ means the position of proton and graphyne in *z* direction.

Supplementary Figures

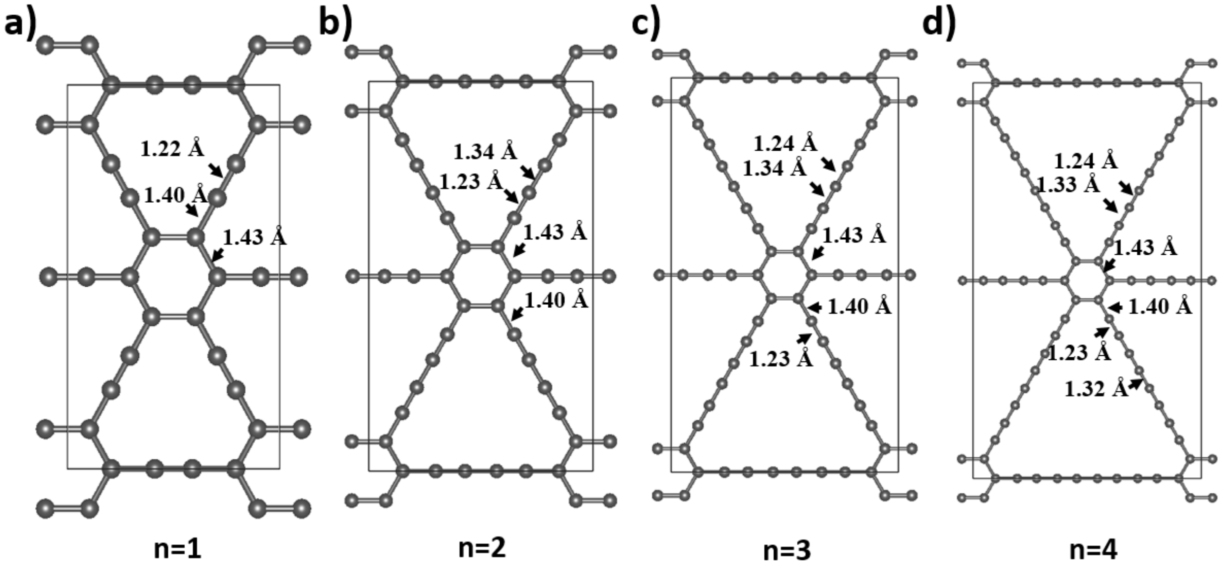

Supplementary Figure 1 Geometrical parameters of graphyne. (a) *n*=1 (b) *n*=2 (c) *n*=3 (d) *n*=4

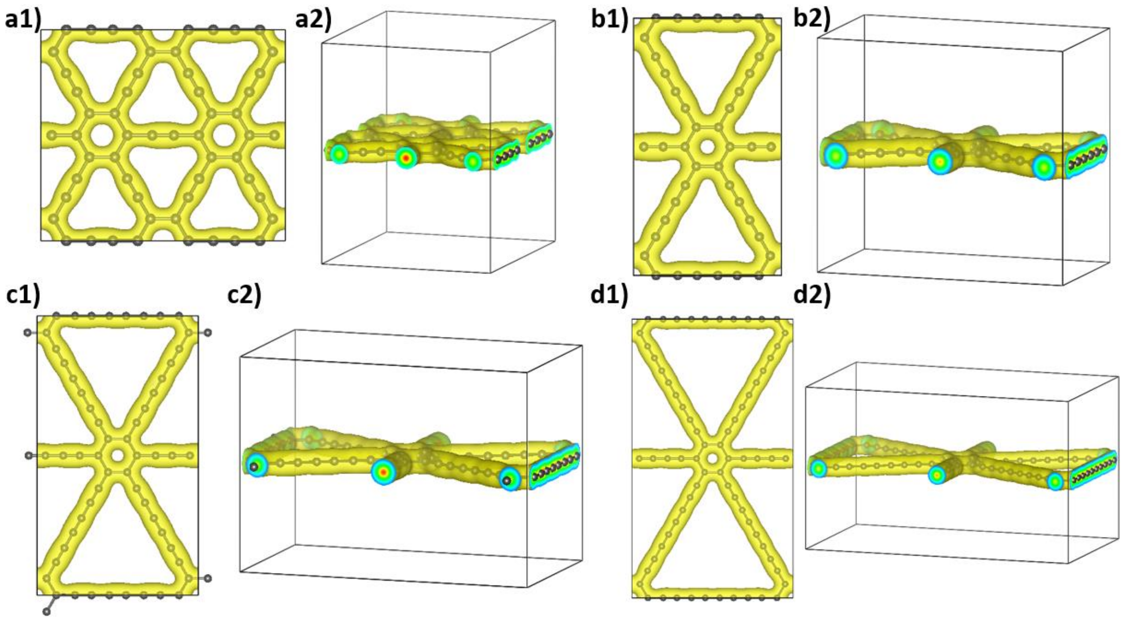

Supplementary Figure 2 Electron distribution of graphyne with (a) n=1 (b) n=2 (c) n=3 and (d) n=4. The isosurface is 0.05 e/bohr

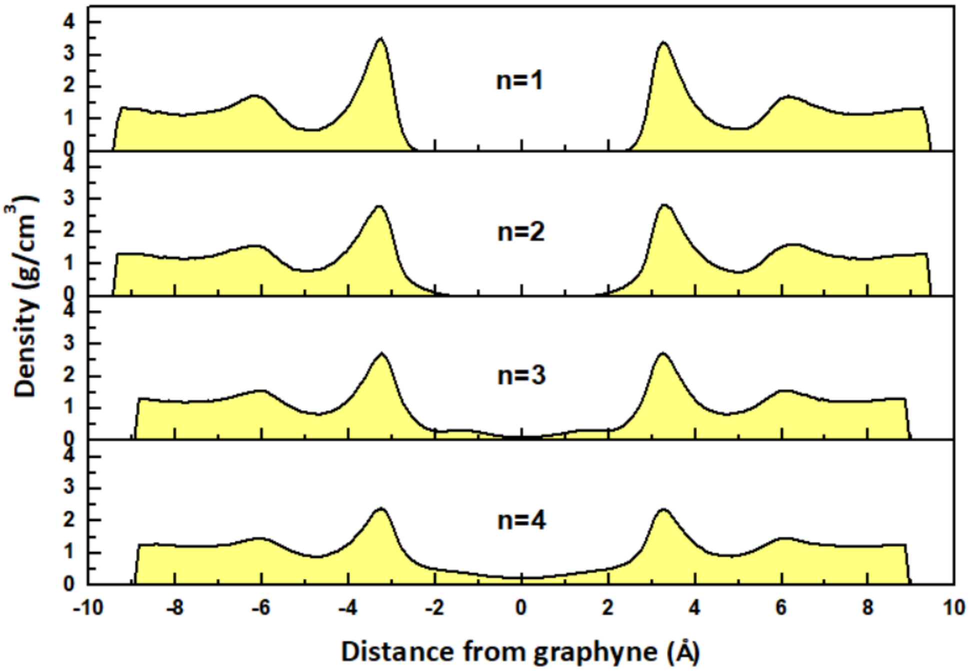

Supplementary Figure 3 Density profiles of water as a function of distance to graphyne with different pore sizes. The data was obtained from classical molecular dynamics simulations for 1 ns for each case.

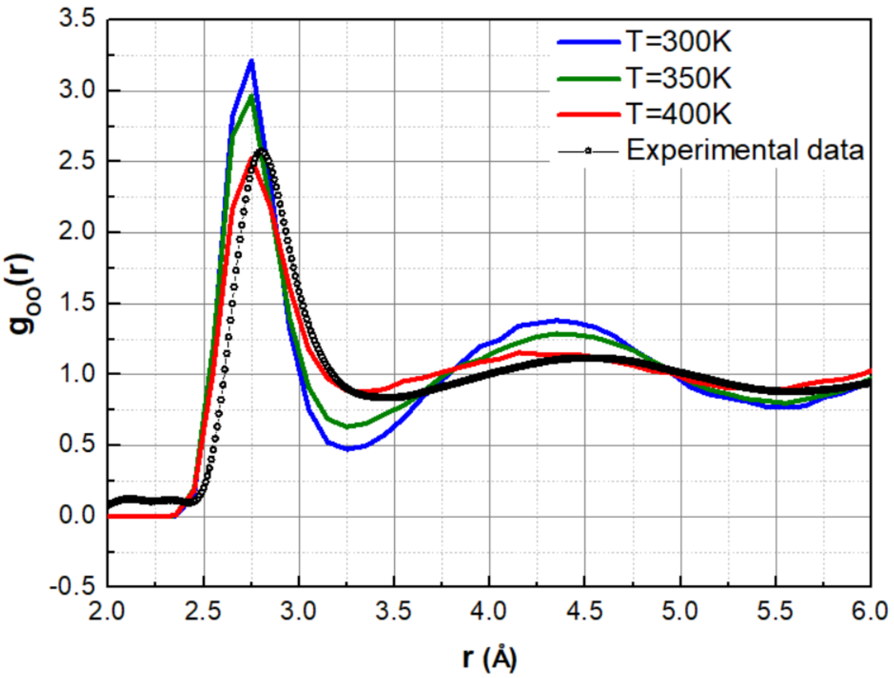

Supplementary Figure 4 Experimental [3] and simulated oxygen-oxygen pair-correlation functions at different simulation temperature.

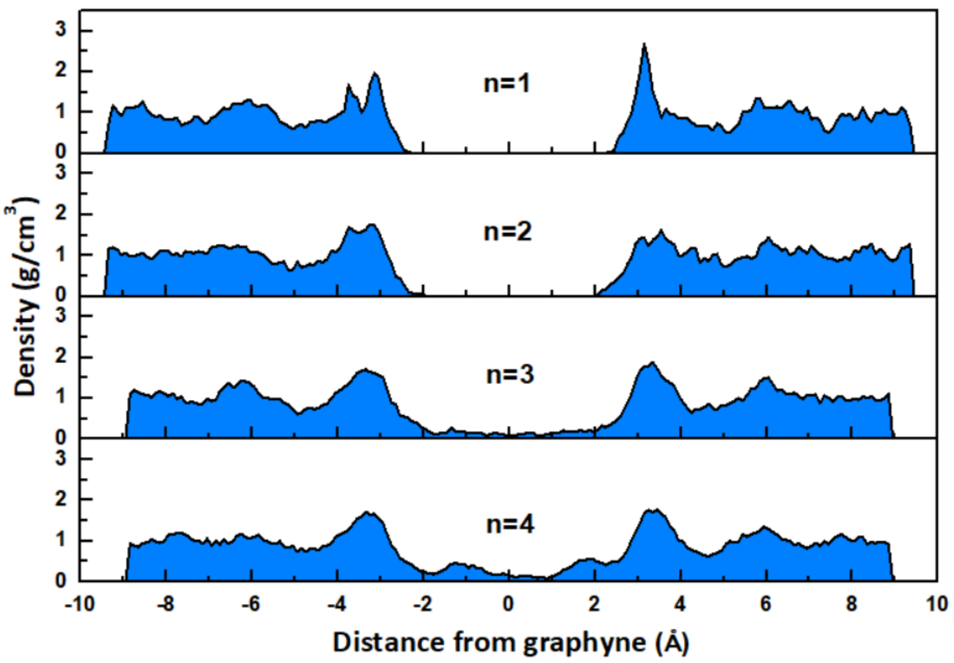

Supplementary Figure 5 Density profiles of water as a function of distance to graphyne with different pore sizes. The data was obtained from ab initio molecular dynamics simulations for 10 ps for each case.

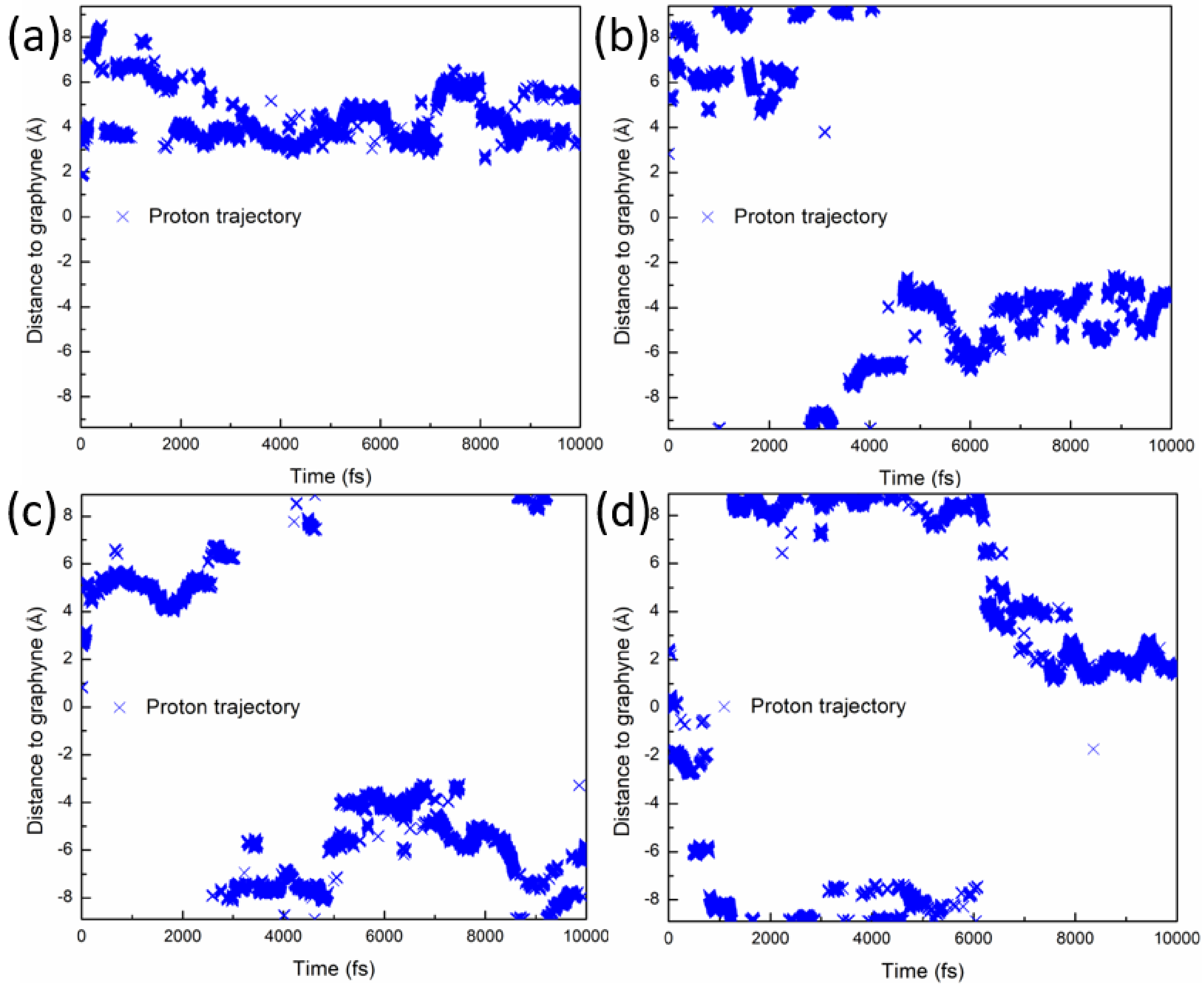

Supplementary Figure 6 The distance between proton and graphyne with (a) n=1 (b) n=2 (c) n=3 and (d) n=4 as a function of time in unbiased ab initio molecular dynamics simulations for 10 ps.

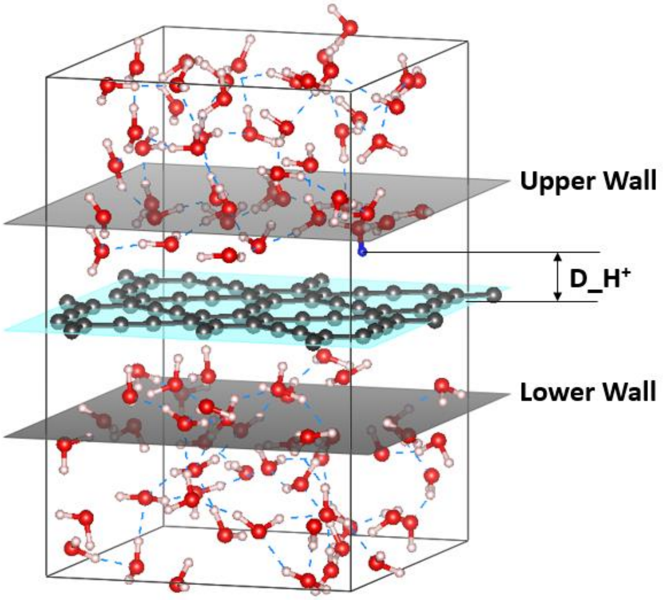

Supplementary Figure 7 Setup for metadynamics simulations of proton penetration across graphyne with *n*=1 and *n*=2 with constraints

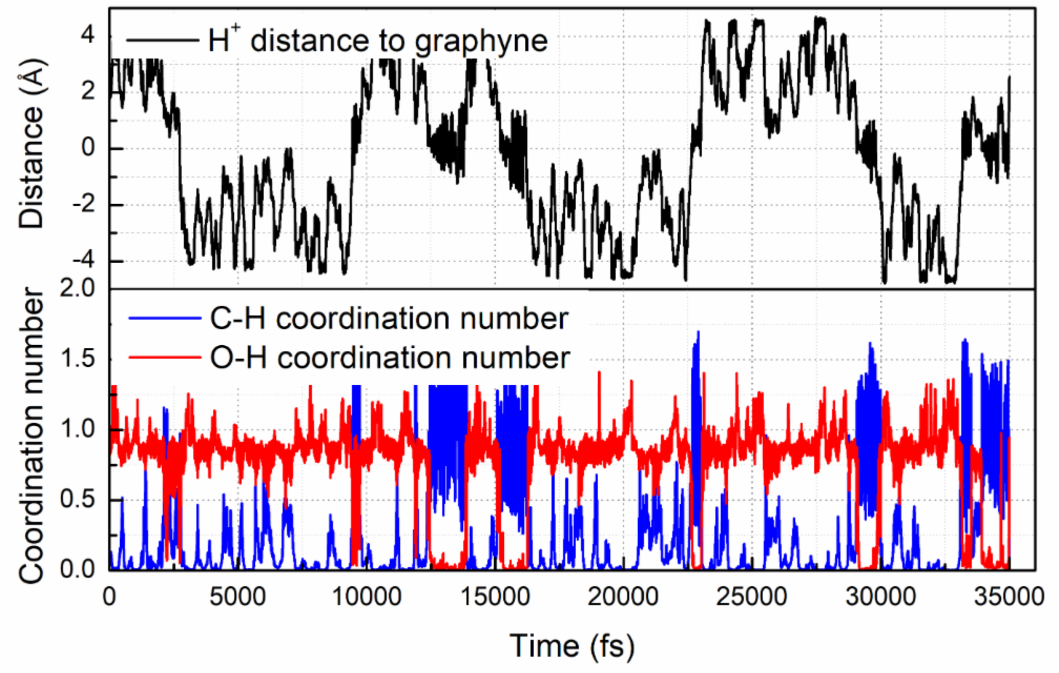

Supplementary Figure 8 Distance between proton and graphyne (n=1), C-H coordination number and O-H coordination number as a function of simulation time in one of the metadyanmics simulations of proton penetration across graphyne (n=1).

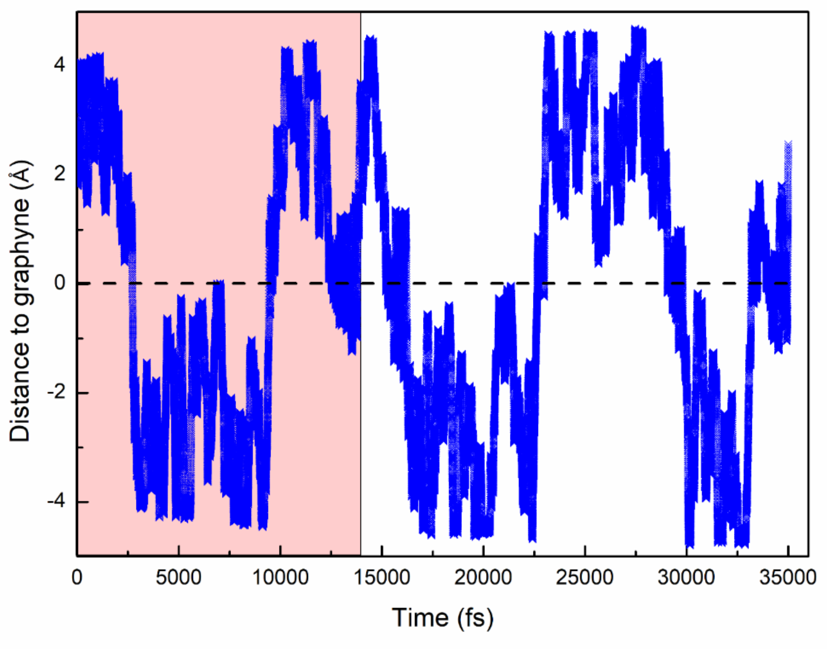

Supplementary Figure 9 Distance between proton and graphyne (*n*=1) as a function of time in one of the metadynamics simulations of proton penetration across graphyne (*n*=1). The pink area represents the data used to construct free energy profile.

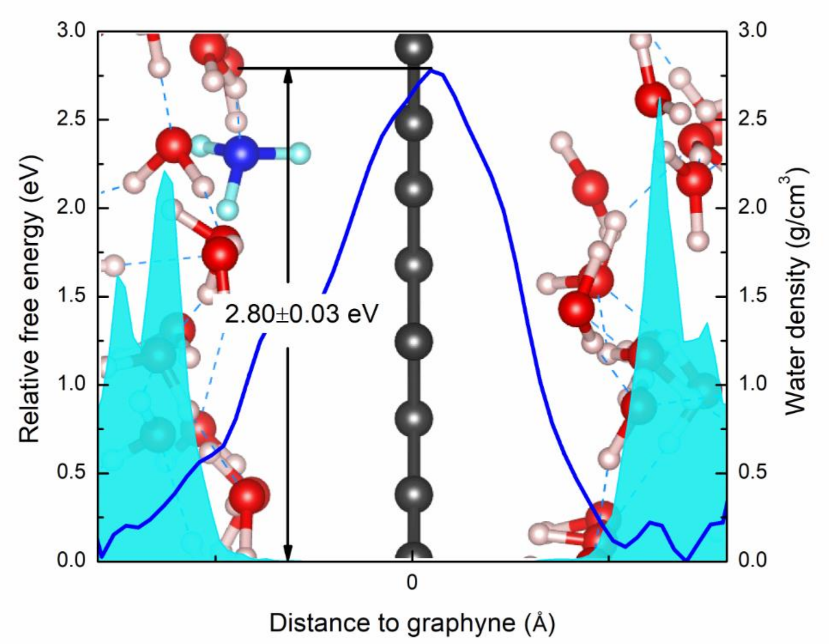

Supplementary Figure 10 Free energy profile of proton and density profile of water as a function of the distance to graphyne (*n*=1). The background shows the initial geometries of the simulation system.

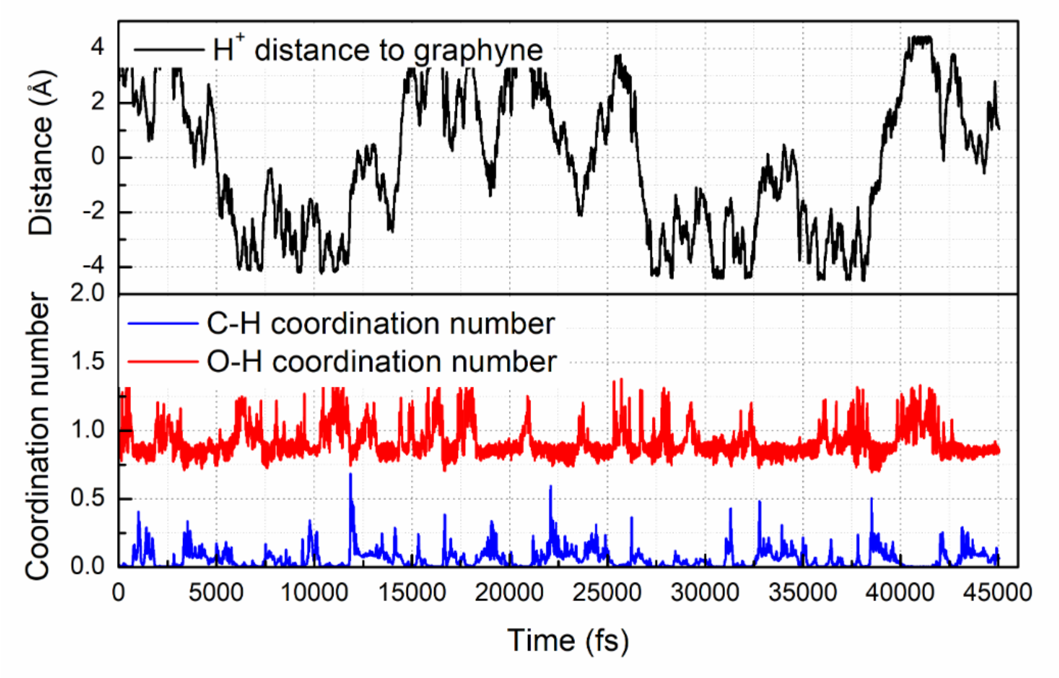

Supplementary Figure 11 Distance between proton and graphyne (n=2), C-H coordination number and O-H coordination number as a function of simulation time in one of the metadyanmics simulations of proton penetration across graphyne (n=2) with constraints.

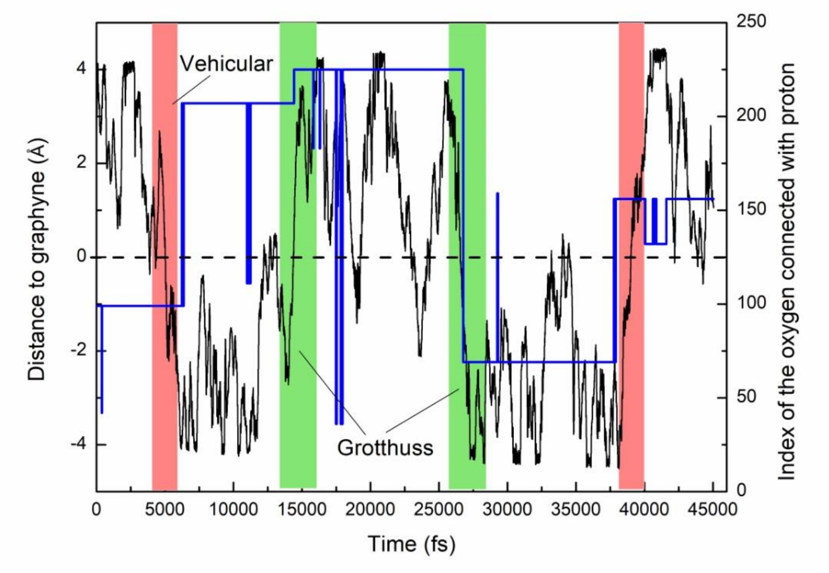

Supplementary Figure 12 Distance between proton and graphyne (n=2) and the index of oxygen atom connected with proton as a function of simulation time. The proton penetration process highlighted in the pink bar occurred via a vehicular mechanism, while the proton penetration process highlighted in the green bar occurred via Grotthuss mechanism.

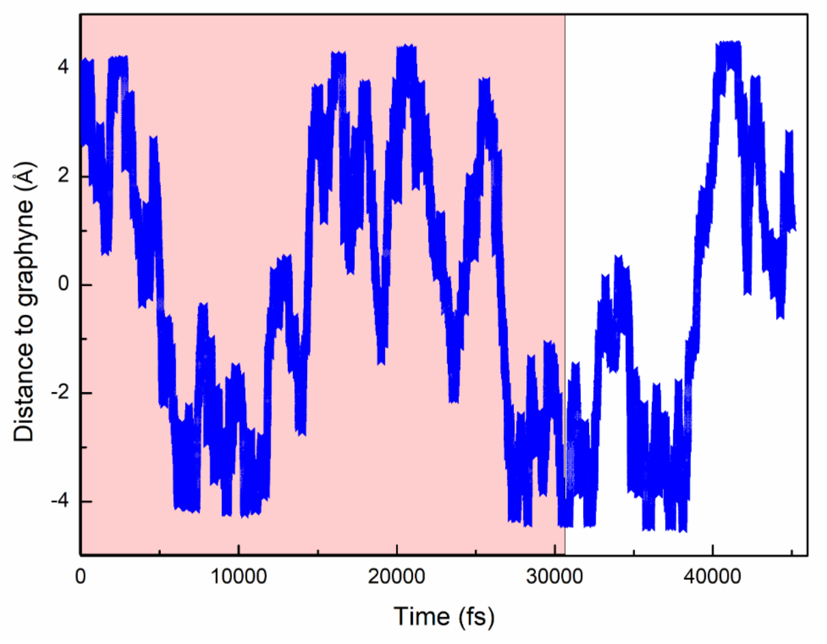

Supplementary Figure 13 Distance between proton and graphyne (n=2) as a function of time in one of the metadynamics simulations of proton penetration across graphyne (n=2) with constraints. The pink area represents the data used to construct free energy profile.

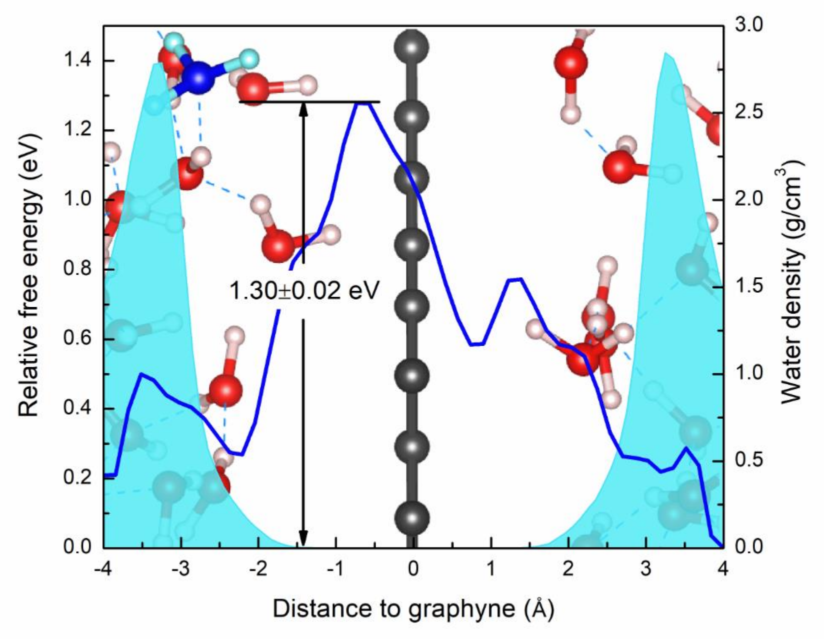

Supplementary Figure 14 Free energy profile of proton and the density profile of water as a function of the distance to graphyne (*n*=2) obtained from the metadynamics simulations with constraints. The background shows the initial geometries of the simulation system.

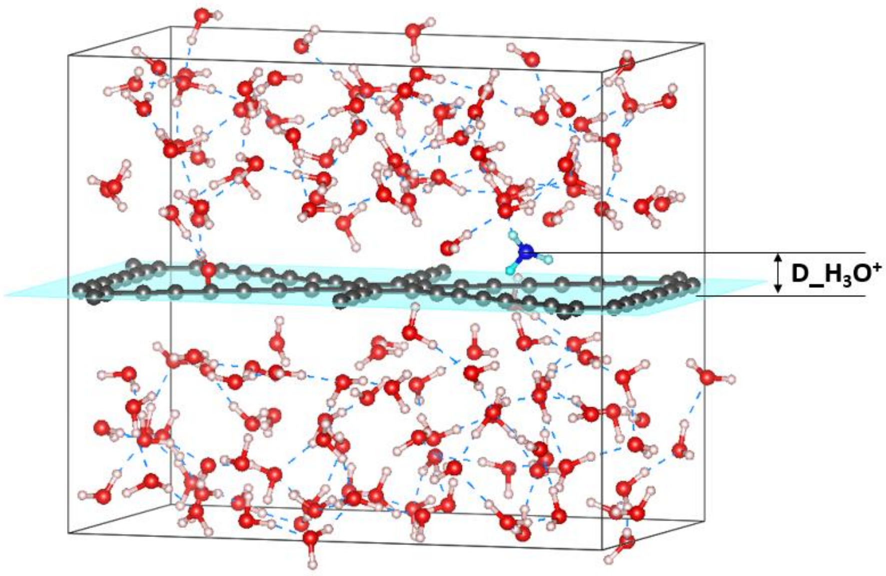

Supplementary Figure 15 Setup for metadynamics simulations of proton penetration across graphyne without constraints for *n*=2, *n*=3 and *n*=4

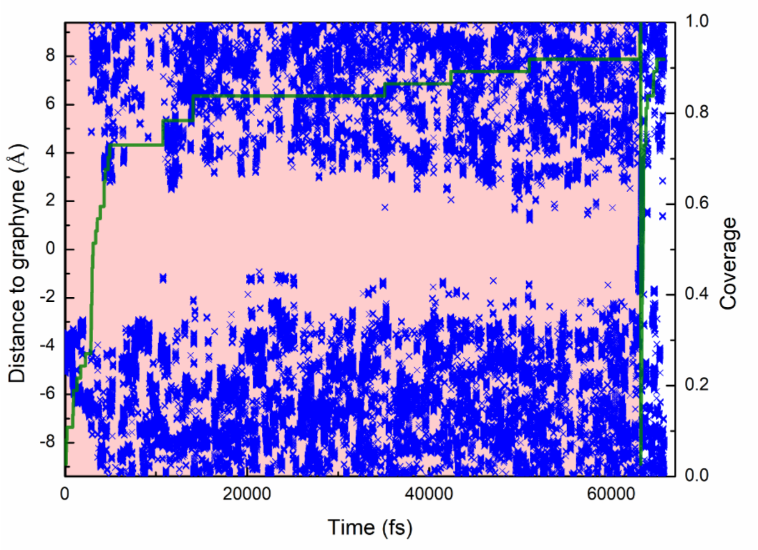

Supplementary Figure 16. Distance between hydronium ion and graphyne (*n*=2) (blue marks) and the coverage of visited hydronium positions in the phase space (green line) as a function of time in the metadynamics simulations of proton penetration across graphyne (*n*=2) without constraints. The coverage was reset once it reaches 1. The pink area represents the data used to construct free energy profile.

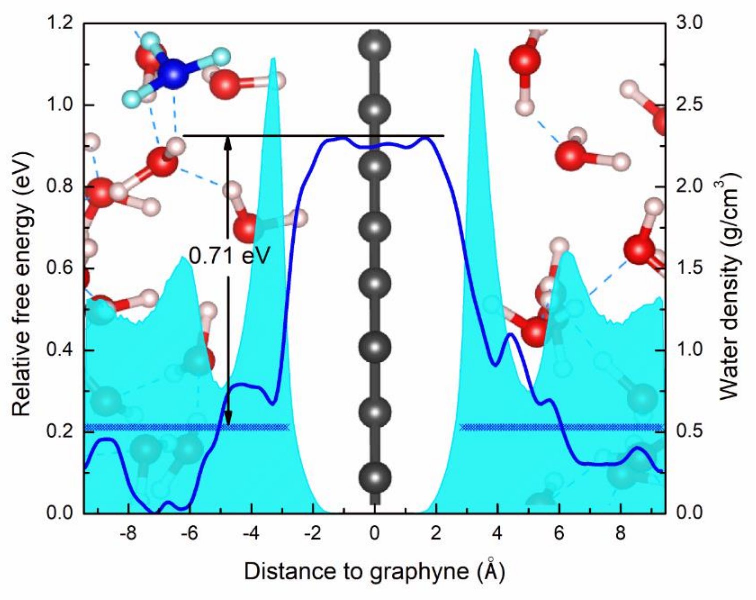

Supplementary Figure 17. Free energy profile of hydronium ion and density profile of water as a function of distance to graphyne (*n*=2) obtained from the metadynamics simulation without constraints. The background shows the initial geometry of the simulation system.

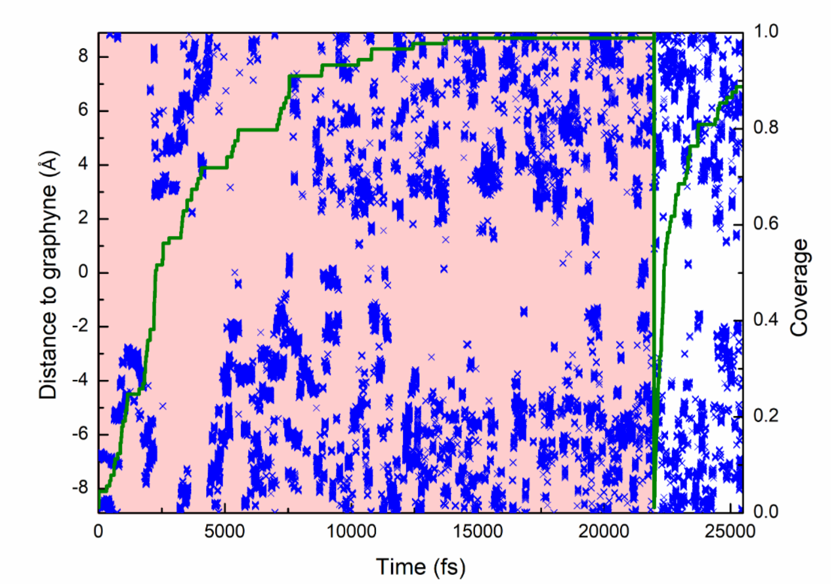

Supplementary Figure 18 Distance between hydronium ion and graphyne (*n*=3) (blue marks) and the coverage of visited hydronium positions in the phase space (green line) as a function of time in one of the metadynamics simulations of proton penetration across graphyne (*n*=3). The coverage was reset once it reaches 1.The pink area represents the data used to construct free energy profile.

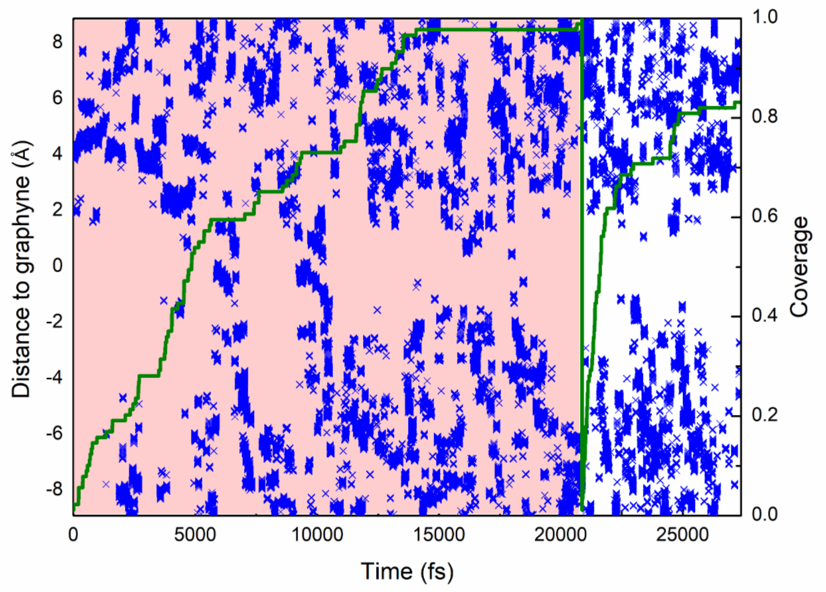

Supplementary Figure 19 Distance between hydronium ion and graphyne (*n*=4) (blue marks) and the coverage of visited hydronium positions in the phase space (green line) as a function of time in one of the metadynamics simulations of proton penetration across graphyne (*n*=4). The coverage was reset once it reaches 1.The pink area represents the data used to construct free energy profile.

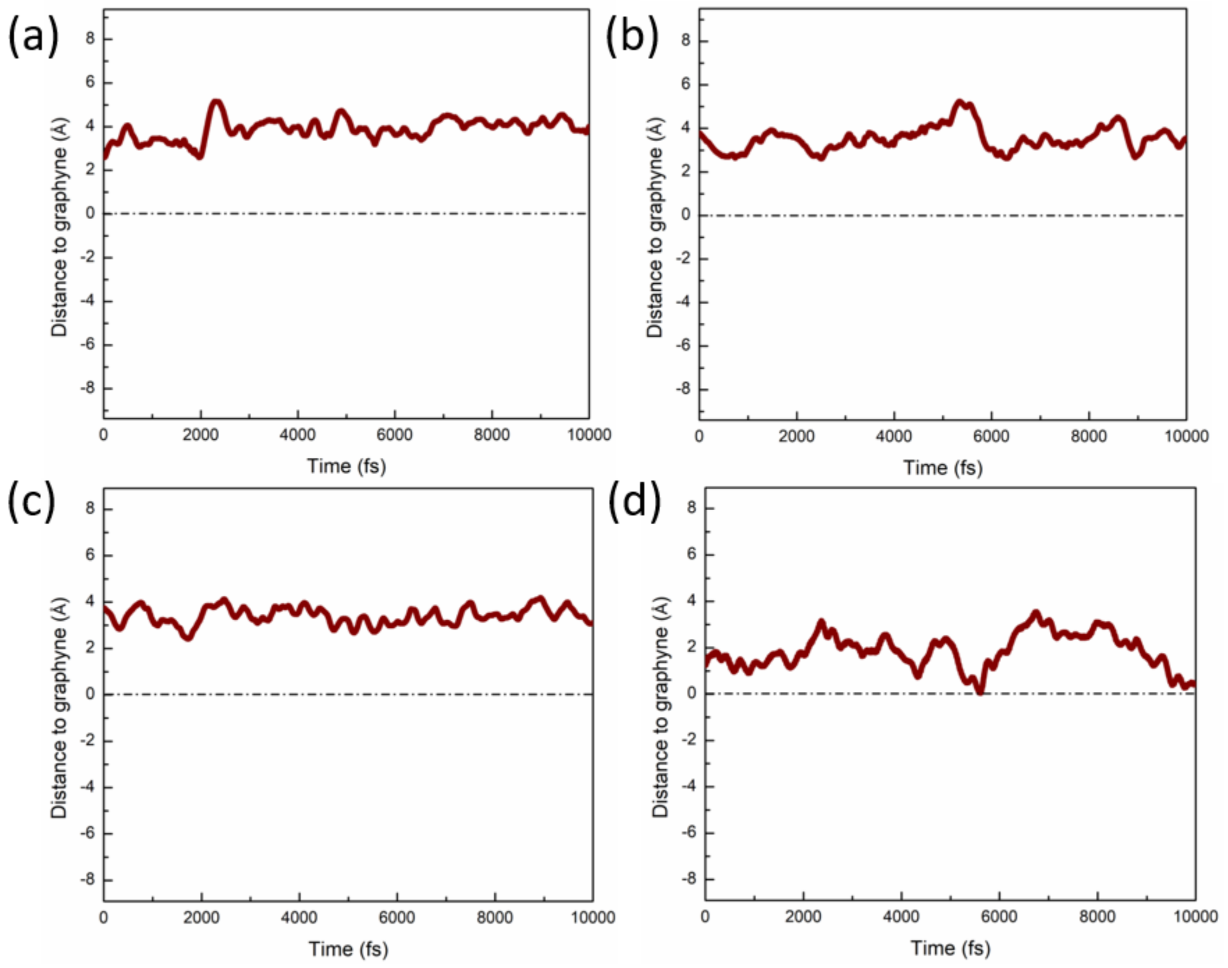

Supplementary Figure 20 The distance between methanol and graphyne with (a) *n*=1 (b) *n*=2 (c) *n*=3 and (d) *n*=4 as a function of time in unbiased *ab initio* molecular dynamics simulations for 10 ps.

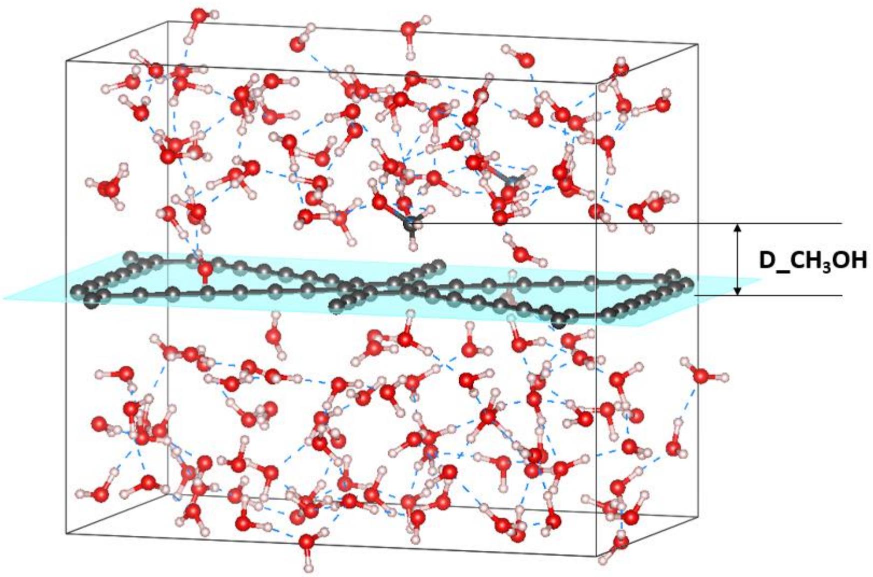

Supplementary Figure 21 Setup for metadynamics simulations of methanol penetration across graphyne with n=3 and n=4

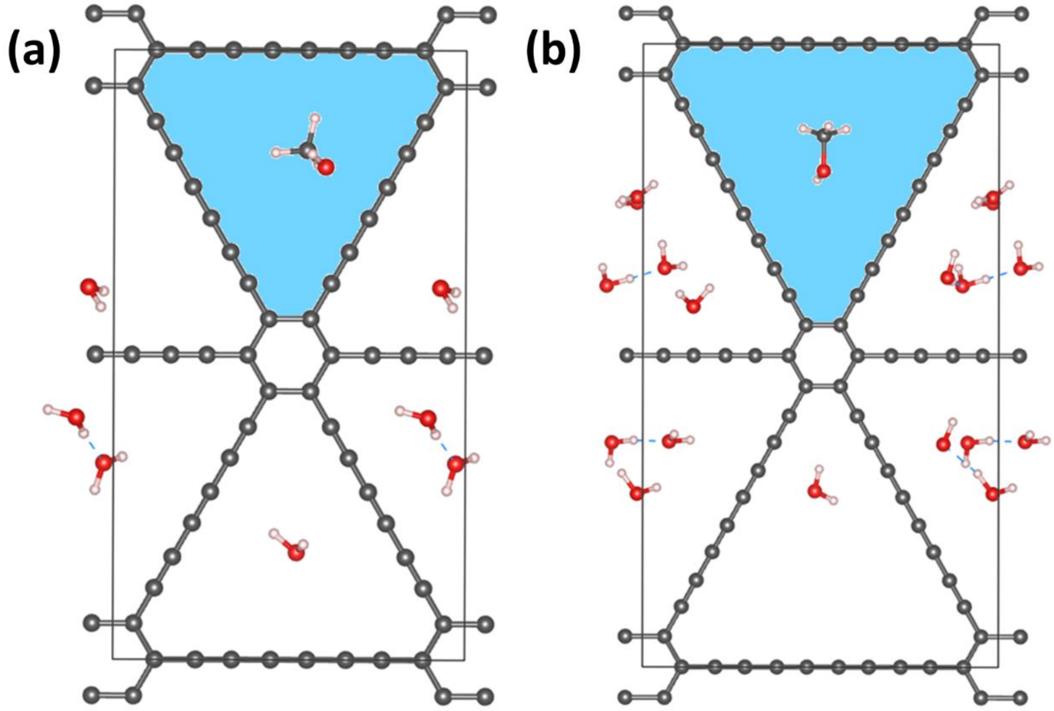

Supplementary Figure 22. Snapshot of methanol penetration across graphyne (a) *n*=3 and (b) *n*=4

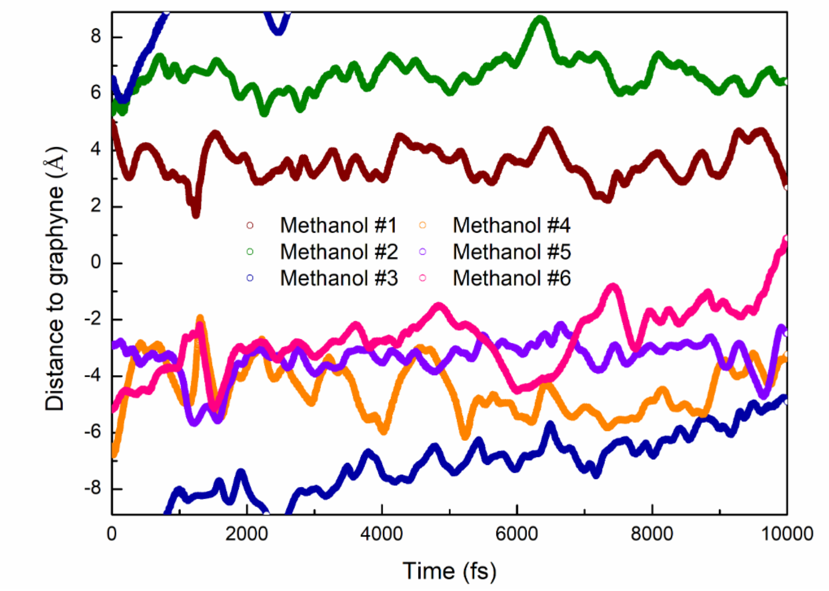

Supplementary Figure 23 Distance between methanol molecules and graphyne (*n*=3) as a function of time in unbiased ab initio molecular dynamics simulations for 10 ps.

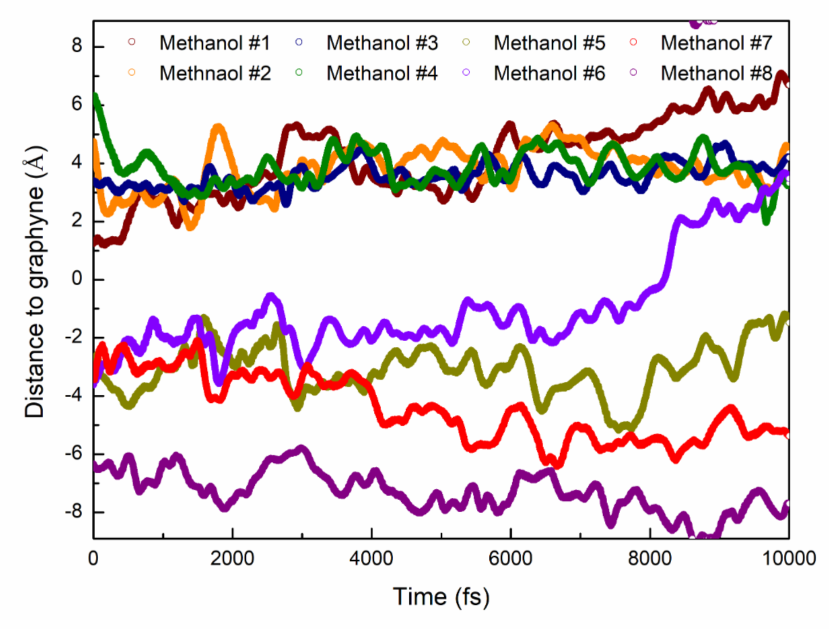

Supplementary Figure 24 Distance between methanol molecules and graphyne (*n*=4) as a function of time in unbiased ab initio molecular dynamics simulations for 10 ps.

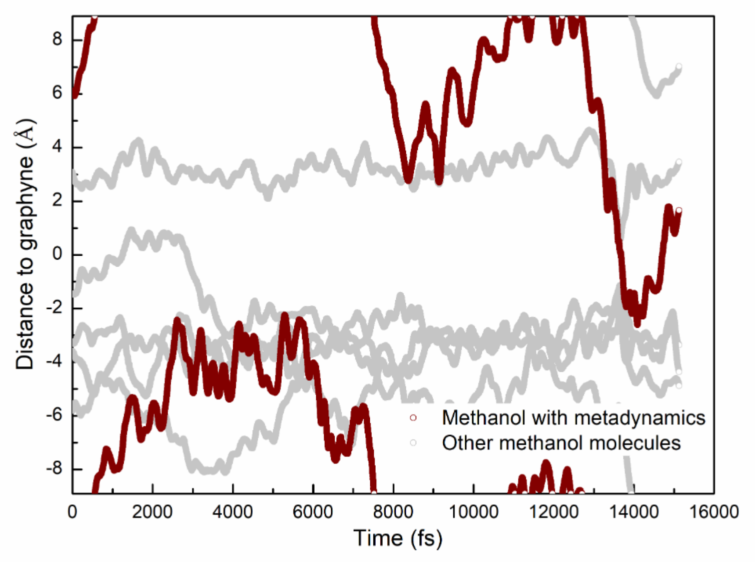

Supplementary Figure 25 Distance between methanol molecules and graphyne (n=3) as a function of time in the metadynamics simulation of methanol penetration across graphyne (n=3).

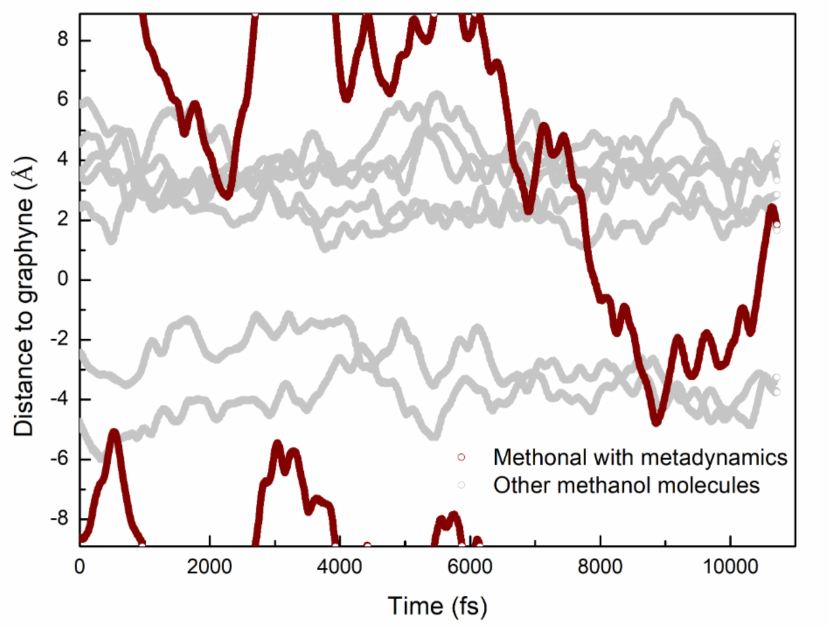

Supplementary Figure 26 Distance between methanol molecules and graphyne (*n*=4) as a function of time in the metadynamics simulation of methanol penetration across graphyne (*n*=4).

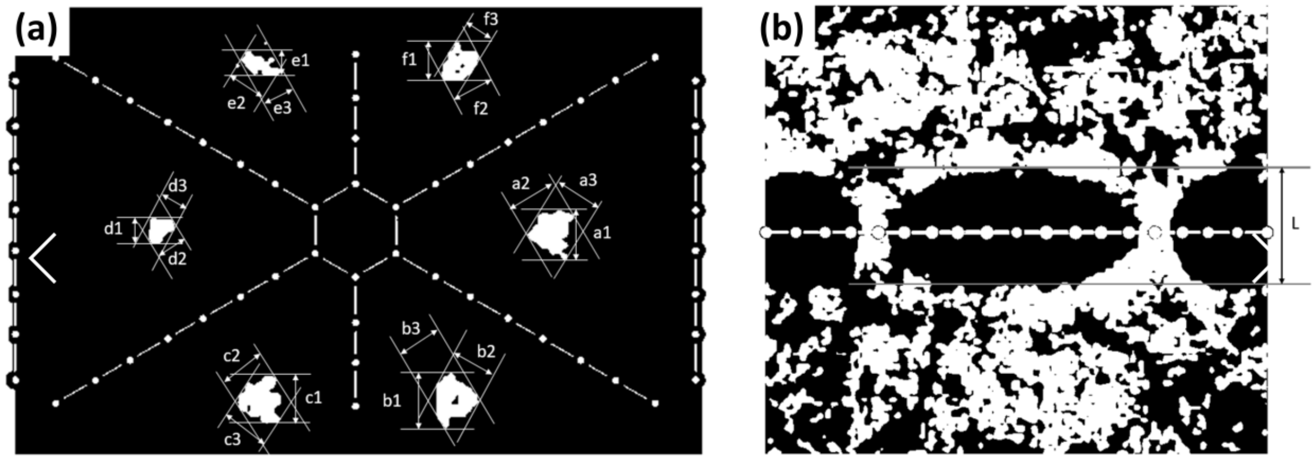

Supplementary Figure 27 Parameters used to estimate the proton conductivity across graphyne (n=3)

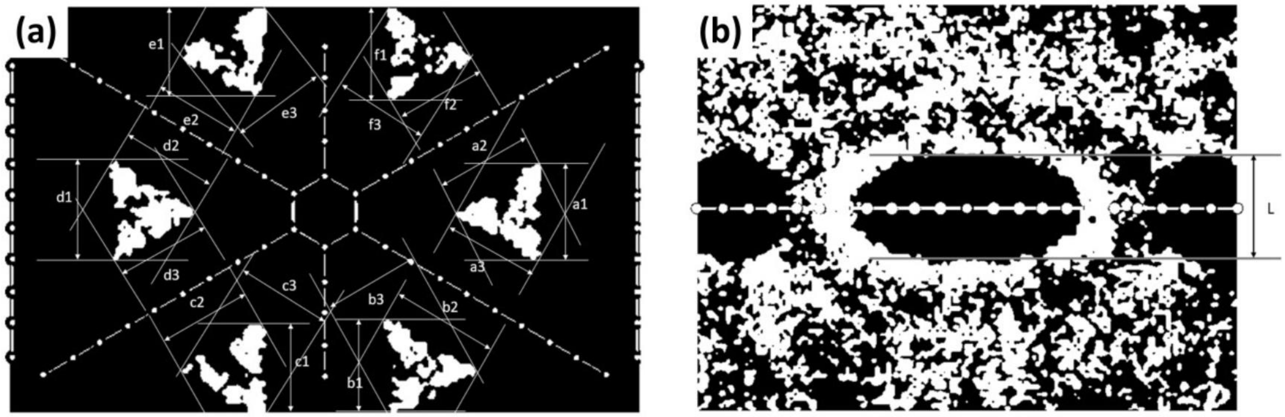

Supplementary Figure 28 Parameters used to estimate the proton conductivity across graphyne (*n*=4)

Supplementary Tables

Supplementary Table 1 Penetration energy barriers of different species across graphyne in vacuum environment with different pore sizes

	n=1	*n*=2	*n*=3	*n*=4
$E_b(H^+)$/eV	N.A.	N.A.	N.A.	N.A.
$E_b(H_2O)$/eV	3.98	0.68	N.A.	N.A.
$E_b(H_3O^+)$/eV	4.35	N.A.	N.A.	N.A.
$E_b(CH_3OH)$/eV	6.62	1.02	N.A.	N.A.

Supplementary Table 2 Simulation setup for graphyne in aqueous environment with different pore sizes

	n=1	*n*=2	*n*=3	*n*=4
a(Å)	13.7773	9.4593	12.0300	14.6002
b(Å)	11.9231	16.3906	20.8500	25.2913
c(Å)	18.7385	18.8021	17.8090	17.8036
N_{water}	83	78	126	185
ρ_{water}(g/cm^3)	0.8060	0.7998	0.8431	0.8411

Supplementary Table 3 length of the triangular pores occupied by aqueous phase in graphyne (*n*=3)

lable	a1	a2	a3	b1	b2	b3	c1	c2	c3	a(average)
length (Å)	1.55	1.50	1.44	1.72	1.33	1.34	1.51	1.39	1.48	1.23
lable	d1	d2	d3	e1	e2	e3	f1	f2	f3	
length (Å)	0.78	0.94	0.87	0.76	1.17	0.95	1.18	1.29	0.90	

Supplementary Table 4 length of the triangular pores occupied by aqueous phase in graphyne (*n*=4)

lable	a1	a2	a3	b1	b2	b3	c1	c2	c3	a(average)
length (Å)	3.87	3.88	3.84	3.69	3.82	3.83	3.53	3.74	3.84	3.76
lable	d1	d2	d3	e1	e2	e3	f1	f2	f3	
length (Å)	4.02	3.83	3.81	3.57	3.47	3.83	3.74	3.63	3.78	

Supplementary Table 5 Deposition pace convergence test for proton penetration across graphyne (*n*=1) with constraints

Pace (fs)	5	10	20
Energy barrier (eV)	3.40	2.83	2.71

Supplementary Table 6 Calculated proton penetration energy barriers with different initial geometries (n=1) with constraints

Initial geometry	#1	#2	#3	Average
Energy barrier (eV)	2.83	2.80	2.77	2.80±0.03

Supplementary Table 7 Deposition pace convergence test for proton penetration across graphyne (*n*=2) with constraints

Pace (fs)	5	10	20
Energy barrier (eV)	1.66	1.29	1.29

Supplementary Table 8 Calculated proton penetration energy barriers with different initial geometries (*n*=2) with constraints

Initial geometry	#1	#2	#3	Average
Energy barrier (eV)	1.29	1.28	1.32	1.30±0.02

Supplementary Table 9 Deposition pace convergence test for proton penetration across graphyne (*n*=3)

Pace (fs)	10	20	40
Energy barrier (eV)	0.42	0.25	0.25

Supplementary Table 10 Calculated proton penetration energy barriers with different initial geometries (*n*=3)

Initial geometry	#1	#2	#3	Average
Energy barrier (eV)	0.25	0.21	0.34	0.27±0.07

Supplementary Table 11 Deposition pace convergence test for proton penetration across graphyne (*n*=4)

Pace (fs)	10	20	40
Energy barrier (eV)	0.21	0.17	0.16

Supplementary Table 12 Calculated proton penetration energy barriers with different initial geometries (*n*=4)

Initial geometry	#1	#2	#3	Average
Energy barrier (eV)	0.17	0.20	0.21	0.19±0.02

Supplementary Table 13 Deposition pace convergence test for methanol penetration across graphyne (*n*=3)

Pace (fs)	5	10	20
Energy barrier (eV)	1.03	0.80	0.82

Supplementary Table 14 Calculated methanol penetration energy barriers with different initial geometries (*n*=3)

Initial geometry	#1	#2	#3	Average
Energy barrier (eV)	0.80	0.81	0.82	0.82±0.02

Supplementary Table 15 Deposition pace convergence test for methanol penetration across graphyne (n=4)

Pace (fs)	5	10	20
Energy barrier (eV)	1.14	0.81	0.80

Supplementary Table 16 Calculated methanol penetration energy barriers with different initial geometries (n=4)

Initial geometry	#1	#2	#3	Average
Energy barrier (eV)	0.81	1.02	0.86	0.90±0.11

Supplementary Table 17 Parameters adopted for the production run of metadynamics simulations

	Pace (fs)	Height (KJ/mol)	Sigma (Å)
Proton penetration across graphyne (n=1) with constraints	10	5	0.25
Proton penetration across graphyne (n=2) with constraints	10	2	0.25
Proton penetration across graphyne (n=2) without constraints	20	0.5	0.25
Proton penetration across graphyne (n=3)	20	0.5	0.25
Proton penetration across graphyne (n=4)	20	0.5	0.25
Methanol penetration across graphyne (n=3)	10	2	0.25
Methanol penetration across graphyne (n=4)	10	2	0.25

Supplementary References

[1] Park, J. M., Laio, A., Iannuzzi, M. & Parrinello, M. Dissociation mechanism of acetic acid in water. *J. Am. Chem. Soc.* **128**, 11318-11319 (2006).

[2] Zhang, C. et al. Water at hydrophobic interfaces delays proton surface-to-bulk transfer and provides a pathway for lateral proton diffusion. *Proc. Natl. Acad. Sci*. **109**, 9744-9749 (2012).

[3] Hura, G., Sorenson, J. M., Glaeser, R. M. & Head-Gordon, T. A high-quality x-ray scattering experiment on liquid water at ambient conditions. *J. Chem. Phys*. **113**, 9140-9148 (2000).
